# Assessment of the potential public health impact of Herpes Zoster vaccination in Germany

**DOI:** 10.1080/21645515.2017.1345399

**Published:** 2017-07-14

**Authors:** Desmond Curran, Desirée Van Oorschot, Lijoy Varghese, Lidia Oostvogels, Tomas Mrkvan, Romulo Colindres, Alfred von Krempelhuber, Anastassia Anastassopoulou

**Affiliations:** a GSK, Wavre, Belgium; b GSK, Singapore; c GSK, München, Germany

**Keywords:** Herpes Zoster, Public Health Impact, Vaccination, Number Needed to Vaccinate

## Abstract

The aim of this study was to compare the public health impact of introducing 2 Herpes Zoster (HZ) vaccines, Zoster Vaccine Live (ZVL) versus a non-live adjuvanted subunit candidate vaccine (HZ/su), in the German population aged 50+ years split into 3 age cohorts, i.e. 50–59, 60–69 and 70+ years, respectively. A multi-cohort static Markov model was developed following age cohorts over their lifetime. Demographic data were obtained from the German federal statistical office. HZ incidence and the proportion of HZ individuals developing post-herpetic neuralgia (PHN) were derived from German specific sources. Age-specific vaccine efficacy and waning rates were based on published clinical trial data. Vaccine coverage for both vaccines was assumed to be 40%, with compliance of the second dose of the HZ/su vaccine of 70%. Sensitivity analyses were performed to assess the robustness of the results. It was estimated that, over the remaining lifetime since vaccination, the HZ/su vaccine would reduce the number of HZ cases by 725,233, 533,162 and 486,794 in the 3 age cohorts, respectively, compared with 198,477, 196,000 and 104,640, using ZVL. The number needed to vaccinate (NNV) to prevent one HZ case ranged from 8 to 11 using the HZ/su vaccine compared with 20 to 50 using ZVL. Corresponding NNV to prevent one PHN case ranged from 39 to 53 using the HZ/su vaccine compared with 94 to 198 using ZVL. Due to the higher, sustained vaccine efficacy, the candidate HZ/su vaccine demonstrated superior public health impact compared with ZVL.

## Introduction

Herpes zoster (HZ) results from a reactivation of latent varicella-zoster virus (VZV) infection, which is believed to occur when VZV-specific cell-mediated immunity (CMI) falls below a critical threshold, either because of aging or immunosuppression.^1^ The majority of older adults are at risk for HZ; for example, 99.5% of the United States (US) population 40+ years of age have been infected with wild-type VZV and are, thus, at risk of developing HZ.^2^ It is estimated that 30% of people will develop HZ during their life time while the lifetime risk increases to 50% in individuals who live beyond 85 y of age.^3^

The acute phase of HZ disease is generally characterized by a unilateral vesicular skin rash in the affected dermatome, which may occur anywhere on the body but most commonly presents on the trunk.^4^ Acute HZ pain has been ranked as more intense than post-surgical or labor pain.^5^ Pain that continues after the rash has healed is termed postherpetic neuralgia (PHN, often defined as pain persisting or appearing 90 d after rash onset), a chronic neuropathic pain syndrome.^6^ Non-PHN complications such as ophthalmic, neurological, visceral and cutaneous HZ are also frequent especially during the acute phase of HZ, and may lead to long-term physical impairment.^7-9^ Although HZ-related mortality is infrequent, the mortality rate does increase with age to approximately 3.86/100,000 person years in the German population aged 90+.^10^

HZ poses a considerable burden on the health care system in Germany both in terms of outpatient and inpatient services.^10^ It was estimated that 306,511 individuals developed HZ annually in Germany based on data from 2007–2008^10^ resulting in a total annual burden to society of approximately €182 million.^11^ On average, an HZ patient taking sick-leave stayed off work for 12.5 d and for, approximately, 2 months in those individuals with PHN.^12^ With the increasing aging of the population the burden related to HZ is likely to increase further.^13^

A one-dose vaccine produced by Merck (Zostavax), referred to as Zoster Vaccine Live (ZVL) is a live vaccine, that utilizes the same Oka strain as the one used in varicella vaccines but at a higher potency, was developed to prevent HZ and PHN.^14^ In the Shingles Prevention Study (SPS) pivotal phase III clinical trial, which was performed in a population of individuals aged 60 y and over, this vaccine reduced the overall incidence of HZ by 51.3% and the overall incidence of PHN by 66.5%.^14^ While this vaccine demonstrated efficacy results against HZ of 69.8% in individuals 50–59 y of age,^15^ the vaccine efficacy against HZ and PHN reduced to 18.3% and 39.5%, respectively in individuals aged 80+.^14,16^

A non-live adjuvanted subunit candidate vaccine (herpes zoster subunit – HZ/su) developed by GSK combines VZV glycoprotein E (gE) and the AS01_B_ adjuvant system. The HZ/su vaccine is developed to be administered in a 2-dose schedule, as 2 doses produced a 3-fold higher gE-specific CMI response than one dose.^1^ Two pivotal phase III clinical trials, ZOE-50 and ZOE-70 (in individuals aged 50+ and 70+, respectively), have been conducted with this vaccine. HZ/su reduced the overall incidence of HZ by 97.2% (ZOE-50) and 91.3% (pooled analysis in subjects 70 y and above from both studies).^17,18^ Vaccine efficacy against PHN was 88.8% in participants 70+ years.^18^ No individual aged 50–69 developed PHN in the vaccine arm of the ZOE-50 clinical trial. The design of the ZOE studies was very close to the SPS study design allowing descriptive comparisons of the results. Some differences did exist, in particular with respect to age groups participating, and while the SPS study included US sites only, the ZOE studies were conducted in 18 countries worldwide. HZ/su avoids the risk of disease resulting from replication of the vaccine virus; in contrast, ZVL is contraindicated for use in immunosuppressed or immunodeficient individuals in whom administration of ZVL may result in disseminated disease.^16, 19^

ZVL was initially licensed in the US and Europe in 2006. No national recommendation has been made in Germany; however, scientific recommendations were made in some federal states, i.e., Saxonia (2013), Thuringia (2014) and Brandenburg (2015). These scientific recommendations do not imply reimbursement although such recommendations play an important role to initiate reimbursement discussions. For the HZ/su vaccine, regulatory submissions are under review in Europe, the US, Canada and Japan.

In Germany, it was demonstrated in 2013 that the most cost-effective strategy for vaccination with ZVL, using a price of €140.48, was at the age of 60 y yielding an incremental cost-effectiveness ratio of €28,146 per quality-adjusted life-year (QALY) gained.^11^ Cost-effectiveness analyses of the HZ/su vaccine are not available for Germany yet, however, one study by an independent research group in the US suggested that, under conservative assumptions, the HZ/su vaccine had a high probability of offering good value for money.^20^

The scope of the current study is to provide an analysis of the potential public health impact of the implementation of the HZ/su vaccine in Germany in comparison to “no vaccination” and to vaccination with the currently available ZVL.

## Results

**Figure 1.**
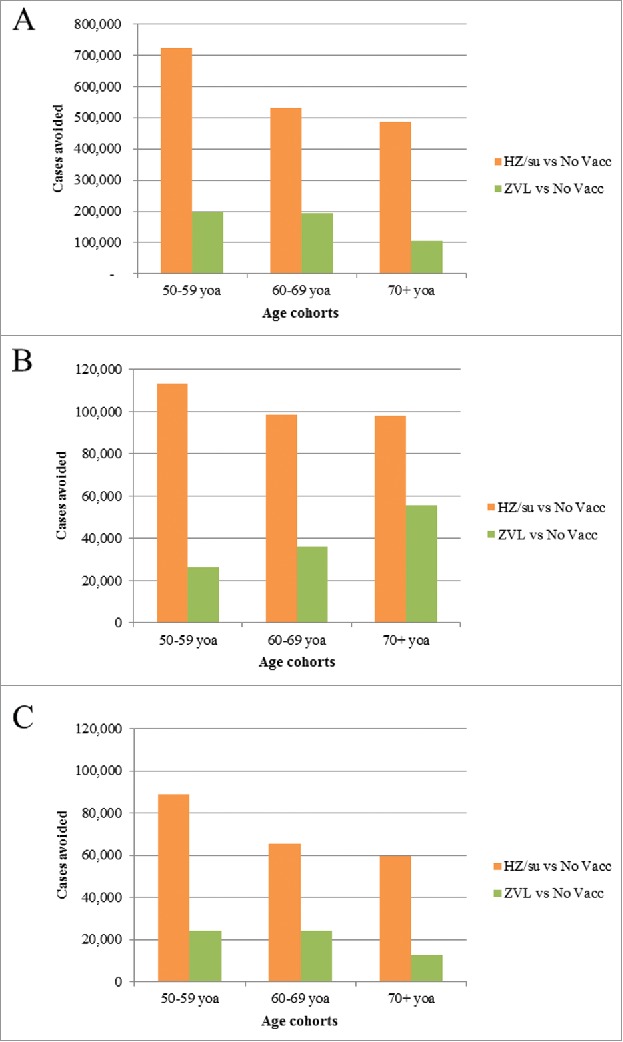
Cases avoided with HZ/su vs. No Vaccination and ZVL vs. No Vaccination by Age Cohort: (A) HZ Cases, (B) PHN Cases, (C) Complications HZ/su: herpes zoster subunit; ZVL: Zoster Vaccine Live; HZ: herpes zoster; PHN: postherpetic neuralgia; No Vacc: No Vaccination; yoa: years of age.

The German population included approximately 13 million, 9.5 million and 13 million individuals in the 3 age cohorts 50–59, 60–69 and 70+ respectively. Figure 1 and Table 1 presents the public health impact over the remaining lifetime using the 3 vaccination strategies, i.e., “no vaccination,” “vaccination with candidate HZ/su” and “vaccination with ZVL.” The results are presented in more granularity in Table S2. In the “no vaccination” strategy, a greater number of HZ cases are projected in the 50–59-year-old age cohort compared with 70+ year old age cohort as these individuals are expected to live longer. On the other hand, more HZ deaths are projected in the 70+-year-old cohort as (i) the risk of HZ death increases with increasing age and (ii) not everyone in the 50–59-year-old cohort will reach the age 70+. Assuming a coverage rate of 40%, and a second dose compliance of 70% for HZ/su, it was estimated that the HZ/su vaccine would reduce the number of HZ cases by 725,223; 533,162, and 486,794 in the 3 cohorts 50–59, 60–69 and 70+, followed over their remaining lifetime, respectively, compared with 198,477; 196,000 and 104,640 in the 3 age cohorts respectively, using ZVL. Consequently, compared with ZVL, the HZ/su vaccine would show an improvement of 265%, 172% and 365% in reducing HZ cases in the 3 age cohorts respectively. Similarly, the HZ/su vaccine would show an improvement of 330%, 173% and 77% in reducing PHN cases and an improvement of 437%, 222% and 423% in reducing number of stays in hospital in the 3 age cohorts, respectively, compared with ZVL.

**Table 1.** Public Health impact of both HZ/su and ZVL under base case assumptions of 40% coverage (HZ/su second dose compliance of 70%) over a lifetime horizon from the age of vaccination.

	Cases	Cases Avoided*	
	No Vaccination	HZ/su	ZVL
Subjects	35,495,639	14,198,256	14,198,256
HZ	9,216,271	1,745,179	499,117
PHN	1,717,545	309,795	117,828
Complications	1,128,993	213,784	61,141
Deaths	3,683	361	32
Hospitalisation	524,602	85,652	19,472
GP Visits	52,169,013	9,388,338	2,478,437

yoa: years of age; HZ: herpes zoster; PHN: postherpetic neuralgia; GP: General Practitioner; HZ/su: herpes zoster subunit; ZVL: Zoster Vaccine Live

*In Vaccinated Subjects compared with no vaccination over the life-time of the respective cohorts

**Figure 2.**
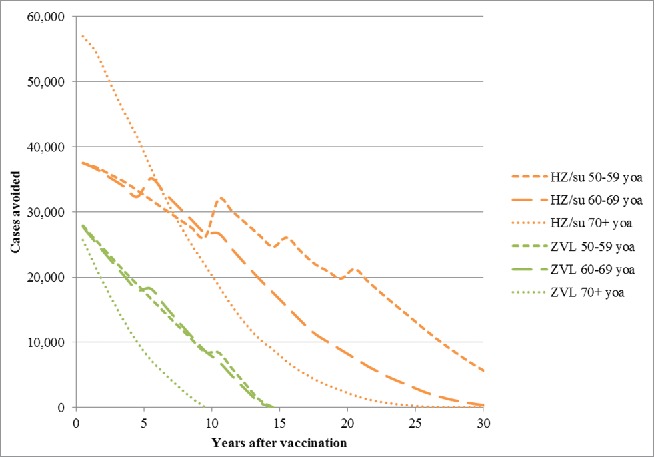
HZ cases avoided with HZ/su vs. No Vaccination and ZVL vs. No Vaccination from the Year of Vaccination by Age Cohort. HZ: herpes zoster; HZ/su: herpes zoster subunit; ZVL: Zoster Vaccine Live. Note incidence is included as an age-specific step function (see Table 4) and this explains the step increases over time, particularly pronounced in the 50–59 y old age group.

Figure 2 presents the number of HZ cases avoided over time since the year of vaccination by age cohort. The greatest benefit occurs in the first few years and decreases over time due to 2 main factors, i.e., waning of efficacy and deaths due to natural causes. For subjects aged 70+ the benefits are observed over a shorter time-frame as compared with younger subjects, i.e., aged <70 y. Due to the higher, sustained vaccine efficacy, the candidate HZ/su vaccine demonstrates superior public health impact compared with ZVL.

Table 2 presents 2 alternative scenarios, i.e., coverage rate of 40% with a compliance rate of 50% and 90% for the second dose of HZ/su. In the scenarios where the second dose compliance was assumed to be 50% and 90%, it was estimated that compared with ZVL, the HZ/su vaccine would show an improvement of approximately 200% and 300%, respectively in reducing HZ cases. The results are presented in more granularity in Table S3.

**Table 2.** Public health impact scenario analysis assuming a coverage of 40% over a lifetime horizon from the age of vaccination (HZ/su second dose compliance of 50% and 90%).

	Cases Avoided*	
	HZ/su : 50% second dose	HZ/su : 90% second dose
Subjects	14,198,256	14,198,256
HZ	1,498,578	1,991,780
PHN	264,082	355,510
Complications	183,576	243,993
Deaths	293	430
Hospitalisation	72,276	99,031
GP Visits	8,008,476	10,768,200

yoa: years of age; HZ: herpes zoster; PHN: postherpetic neuralgia; GP: General Practitioner; HZ/su: herpes zoster subunit.

*In Vaccinated Subjects over the life-time of the respective cohorts

Table 3 presents the number needed to vaccinate (NNV) to prevent one HZ case and to prevent one PHN case, respectively. In the base case 8, 8 and 11 individuals need to be vaccinated with HZ/su to prevent one HZ case in the 3 cohorts aged 50–59, 60–69 and 70+, respectively. The corresponding NNV for ZVL are 27, 20 and 50, in the 3 age cohorts, respectively.

**Table 3.** Number needed to vaccinate to prevent one HZ case and one PHN case.

	HZ/su Second dose compliance			
	50%	70%	90%	ZVL
NNV for HZ				
50–59 yoa	9	8	7	27
60–69 yoa	9	8	7	20
70+ yoa	13	11	10	50
NNV for PHN				
50–59 yoa	56	46	39	198
60–69 yoa	45	39	35	106
70+ yoa	62	53	47	94

yoa: years of age; HZ: herpes zoster; PHN: postherpetic neuralgia; HZ/su: herpes zoster subunit; NNV: Number needed to vaccinate. Note estimated NNV values were rounded up to the nearest integer.

**Figure 3.**
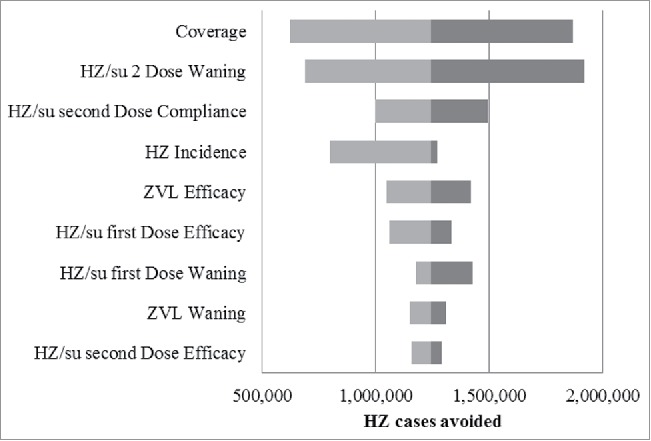
Tornado Diagram: HZ cases avoided with HZ/su compared with ZVL HZ/su: herpes zoster subunit; HZ: herpes zoster. The ranges used for the DSA are detailed in the Table S1.

The results of the deterministic sensitivity analysis (DSA) are summarized in the tornado diagram presented in Fig. 3. In the base case, the HZ/su vaccine resulted in approximately 1.2 million HZ cases more being avoided, over their remaining lifetime, compared with ZVL, in individuals aged 50+ years of age. In all scenarios the candidate HZ/su vaccine resulted in more HZ cases being avoided compared with ZVL. The outcomes were most sensitive to assumptions regarding the vaccine coverage, the 2-dose waning, 2-dose compliance and the incidence rates.

**Figure 4.**
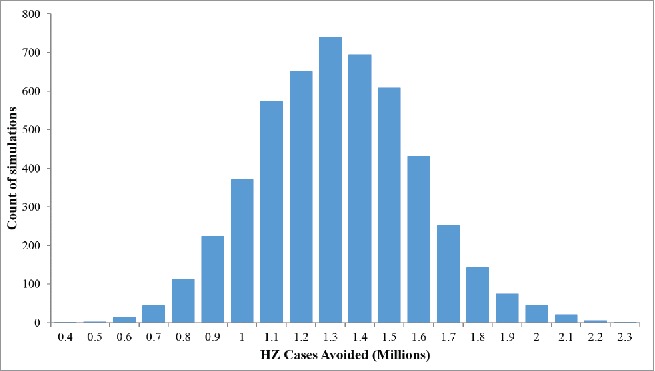
Probabilistic Sensitivity Analysis: HZ cases avoided with HZ/su compared with ZVL (5,000 simulations). HZ/su: herpes zoster subunit; HZ: herpes zoster. The ranges used for the PSA are detailed in the Table S1.

The results of the probabilistic sensitivity analysis (PSA) are summarized in the histogram presented in Fig. 4. In all the 5,000 simulations the candidate HZ/su vaccine resulted in more HZ cases being avoided compared with ZVL, ranging from approximately 0.4 to 2.3 million extra cases avoided with HZ/su compared with ZLV. The number of HZ cases avoided when using HZ/su compared with ZVL, was greater than 0.9 million in more than 90% of simulations.

The scenario analysis presented in Table S4, suggest that in the most optimistic situation regarding efficacy and waning, approximately 2.5 million additional cases of HZ could be avoided using HZ/su compared with ZVL, whereas in the most pessimistic situation regarding efficacy and waning, approximately 0.2 million additional cases of HZ could be avoided using HZ/su compared with ZVL.

## Discussion

In this study we presented the assessment of potential public health impact of both ZVL and the subunit adjuvanted candidate vaccines in reducing the burden associated with HZ. It was estimated that the HZ/su vaccine would reduce the number of HZ cases by approximately 1.75 million, compared with 0.5 million using ZVL, in German adults aged 50+. The number needed to vaccinate (NNV) to prevent one HZ case ranged from 8 to 11 using the HZ/su vaccine compared with 20 to 50 using ZVL.

For consistency, clinical trial data were used to estimate vaccine efficacy for both HZ and PHN of both vaccines. As the HZ/su vaccine is not yet licensed, no effectiveness data are currently available for this vaccine. Effectiveness data have been published for ZVL. The most recent effectiveness estimate with the longest follow-up duration was using data from a Kaiser Permanente Southern California study published by Tseng et al.^21^ In this study, the observed effectiveness of the vaccine against HZ fell from 68.7% (95% CI, 66.3% to 70.9%) in the first year to 4.2% (95% CI, −24.0% to 25.9%) in the eighth year. The decreasing pattern was similar between the younger (aged 60–69 years) and older (age 70+) age cohorts. Fitting a linear estimate to the effectiveness values by year results in an estimated waning of 7.5%, suggesting a more rapid waning of vaccine efficacy against HZ for ZVL than was used in our model.

The vaccine coverage was assumed to be 40%. This assumption was based on other vaccine coverage in adults in Germany and also on a comparison of coverage rates for ZVL in the US and UK.^22^ Coverage rates in the US have been reported to be 23.9% of adults aged 60–64 years, and 14.5% in adults aged 65+ years ^23^ whereas in the UK they have varied between 54.9% and 61.8% in the routine cohort aged 70 y.^24^ As no herd effect is assumed, the results presented here can be easily modified to reflect other coverage rates. For example in Fig. 3, in the base case (i.e., coverage = 40%), 1.2 million more cases are avoided using HZ/su compared with ZVL. When the vaccine coverage is assumed to be 20% and 60%, the corresponding results are 0.6 million and 1.8 million more cases avoided, respectively (see Fig. 3). Similarly, in Table 1, if we assume a coverage of 20% instead of 40%, we can easily calculate that 872,589.5 (= 1,745,179/2) cases of HZ could be prevented by HZ/su under the base-case scenario.

Compliance for the second dose of HZ/su was assumed to be 70% in the base-case analysis. The only data available for second dose compliance for the HZ/su vaccine are coming from the ZOE-50 and ZOE-70 clinical trials where the overall compliance was approximately 95%. When investigating the compliance by study site, the median was 98.4% while the lowest 10^th^, 5^th^ and 1^st^ percentile of compliance was 88.5%, 83.3% and 66.7%, respectively for all sites worldwide. In this study, we also explored the impact of other compliance rates, i.e., 50% and 90%, in the scenario analysis.

The public health impact of HZ/su against no vaccination has been presented for the US, Australia, Germany, the UK and Canada.^25-29^ These studies used varying assumptions regarding incidence, coverage, VE and waning rates. For example, exponential VE waning rates of 2% and 4% were used in the German study.^27^ The VE inputs and assumptions used in this paper were developed using more robust methods and were validated during an advisory board meeting with international experts. Nevertheless, the results of previous studies are consistent with the findings reported here. For example in the Germany study, it was reported that vaccinating 25% of the German adults 50+ years old with the HZ/su vaccine, assuming 100% compliance for the second dose, could potentially prevent between 1.3 – 1.6 million HZ cases compared with a no vaccination strategy, depending on the waning rate assumed. In this study, we further investigated the public health impact of HZ/su compared with the currently available ZVL in the German setting. The sensitivity analysis demonstrated that the results were sensitive to various input parameters, such as coverage, HZ incidence and vaccine parameters. Nevertheless, it was observed that in all the scenarios tested, the HZ/su vaccine provided a greater public health benefit compared with ZVL. Another study in Canada also illustrated the greater public health benefit for the HZ/su vaccine compared with ZVL.^30^ Although, the study used a different model and alternative model assumptions, e.g. regarding efficacy and waning, in the majority of scenarios presented, the HZ/su vaccine demonstrated superior public health impact compared with ZVL.

One limitation of our model is that estimates of VE waning rates, generated from clinical trials where follow-up was limited to under 4 years, were used to project future waning rates. As such there is uncertainty regarding waning rates. Nevertheless, we validated the VE waning rates and upper and lower bounds with a group of international experts. The upper and lower bounds were incorporated in both deterministic and probabilistic sensitivity analysis and scenario. Even under the most conservative vaccine efficacy and waning scenarios, HZ/su was estimated to result in a positive public health impact compared with ZVL. Nonetheless, long-term effectiveness studies will need to be performed to generate VE estimates in a real world setting. Another limitation of this modeling exercise is that we did not adjust for the impact of the current usage of ZVL in the 3 federal states with recommendations for HZ vaccination. Nevertheless, unpublished data suggests that the overall vaccine coverage is less than 1% in German adults aged 50 y and older. As such the impact should be negligible.

This evidence may help clinicians, payers and policy makers in their assessment of the value of vaccination against HZ, not only in Germany but also in other countries where there is an unmet need regarding the prevention of HZ disease.

## Methods

### Mathematical model

**Figure 5.**
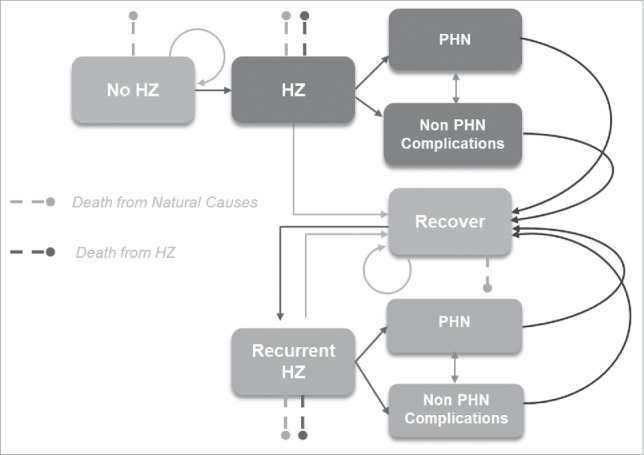
Schematic overview of Markov structure – ZONA model. HZ: herpes zoster; PHN: postherpetic neuralgia.

The ZOster ecoNomic Analysis (ZONA) model (Fig. 5) was developed in MS Excel. It is a static multi-cohort Markov model. Cohorts are split into 5 age groups for people aged 50+ years (i.e., 50–59, 60–64, 65–69, 70–79, 80+). If the “50+ years combined” option is selected, the model assumes that all of the subjects in the 50–59, 60–64, 65–69, 70–79 and 80+ age cohorts are vaccinated, as in a one-off ‘catch-up’ campaign, at an age of 50, 60, 65, 70 and 80 y respectively. The model follows all subjects within a cohort over their remaining life-time from the year of vaccination with annual cycle lengths. As such all subjects remain in their initial cohort and all subsequent events are counted in that cohort only. Three different HZ vaccination strategies are compared; no vaccination (control), vaccination with ZVL, and vaccination with HZ/su. Within each vaccine arm/strategy, individuals can be fully compliant with the vaccine dosing schedule, partially compliant or not vaccinated at all, depending on the corresponding vaccine coverage and compliance rates assumed. An overview of the model structure is presented in Fig. 5. Transition probabilities between the health states HZ, natural death, HZ related deaths, recover, recurrent HZ,.. occur using an annual time step. PHN and NON-PHN complications are health states which occur within a HZ episode and as such occur within this annual time step. Probabilities of moving between health states are derived from Germany specific literature and are age-group specific.

In this analysis 3 age cohorts were considered, i.e., 50–59, 60–69 and 70+ years, i.e., combining results from the 60–64 and 65–69 cohorts and the 70–79 and 80+ cohorts, respectively, for presentation purposes. The age cohorts were selected to capture age-dependent differences in disease incidence, complications, outcomes, costs and potential public health decision making.

### Model inputs

The parameters are divided into 3 distinct sections: demographics, natural history of disease and vaccine efficacy (VE).

#### Demographics

Age-stratified population figures for the year 2015 and all-cause mortality rates were retrieved from the German Federal Statistical Office (Statistisches Bundesamt).^31^

#### Natural history of the disease

Table 4 presents a summary of the Epidemiological inputs included in the model. HZ incidence rates in Germany have been reported in several recent peer-reviewed publications.^10,32-34^ For this analysis, the incidence data of HZ and associated proportion of individuals developing PHN was taken from the recently published manuscript by Hillebrand et al.^33^ The latter study was conducted using data from the German Pharmacoepidemiological Research Database, and assessed claims data for approximately 7 million individuals from 3 statutory health insurances from all geographical regions of Germany. In the absence of data on the recurrence of HZ for Germany, and in alignment with Ultsch et al.,^12^ we assumed that rates of recurrent HZ episodes were the same as the rates of first HZ occurrence based on data from the US.^35^

**Table 4.** Base Case Epidemiological inputs.

Age Groups	Incidence (per 1,000)^33^	PHN*^33^	HZ Mortality Rates*^10^	Hospitalization*^10^	Number of Physician Visits**^38^
50–59 yoa	7.75	13.0%	0.001%	0.023%	4.13
60–64 yoa	10.02	15.4%	0.003%	0.034%	4.94
65–69 yoa	11.44	17.5%	0.005%	0.041%	4.97
70–74 yoa	13.43	19.9%	0.010%	0.057%	5.92
75–79 yoa			0.025%		
80–84 yoa	13.90	20.4%	0.043%	0.081%	6.36
85+ yoa			0.165%		

yoa: years of age; HZ: Herpes Zoster; PHN: postherpetic neuralgia,

* % of HZ cases,

** Mean number of visits per HZ cases

HZ mortality rates were taken from Ultsch et al.^10^ which used the information system of the German Federal Health Monitoring System (FHM) to assess the annual number of HZ-associated deaths.

We included 4 types of non-PHN complications in the analysis, zoster ophthalmicus (5.44%), neurological complications (0.65%), Zoster generalisatus (0.68%) and other complications (5.48%) with estimates taken from Horn et al.^36^ The proportion of individuals developing non-PHN complications was assumed to be consistent across all age groups.^37^

The HZ hospitalisation probabilities were taken from Ultsch et al.,^10^ who used the German FHM data to assess the incidence of HZ leading to hospitalisation in people aged 50+ years. The number of ‘Family Physicians Visits’ were derived from Schiffner-Rohe et al.^38^ who estimated healthcare resource consumption and health care costs of HZ and PHN individuals in Germany on the basis of routine health care data of a German SHI. The number of visits was reported for HZ patients without PHN and additional visits in HZ patients with PHN.

#### Vaccine efficacy

The VE inputs and assumptions for both vaccines were validated during an advisory board meeting with experts in epidemiology, modeling and immunology from the United States, Canada and Germany. The VE of ZVL against HZ and PHN is taken from the SPS and Zoster Efficacy and Safety Study (ZEST),^15^ along with the Short Term Prevention Study (STPS) and Long Term Prevention Study (LTPS) follow-up studies.^14,16,39^ VE of HZ/su against HZ and PHN is taken from the ZOE-50 and ZOE-70 studies.^17, 18^

The efficacy of ZVL was evaluated in 2 phase III clinical trials involving more than 38,000 individuals 60+ years of age (SPS study) and 22,000 individuals 50–59 y of age (ZEST study), respectively.^14,15^ Table 5 presents the overall efficacy against HZ and PHN from the 2 studies. A top-up efficacy was observed during the clinical trials, meaning that the probability of developing PHN was lower in breakthrough HZ cases in the vaccinated group, compared with those individuals with HZ in the placebo group.

**Table 5.** Vaccine efficacy against HZ and PHN.

	Age groups	HZ Efficacy (%)	PHN Efficacy (%)
ZVL ZEST Study^15^			
	50–59 yoa	69.80	NE
ZVL SPS Study^14^			
	60–69 yoa	63.89	65.69
	70–79 yoa	40.85	73.38
	80+ yoa	18.25	39.51
ZOE-50 HZ/su 2-dose^17^			
	Overall 50+ yoa	97.16	100
	50–59 yoa	96.57	NE
	60–69 yoa	97.36	NE
	70+ yoa	97.93	NE
ZOE-70+ HZ/su 2-dose^18^			
	70+ yoa	91.30	88.8
	70–79 yoa	91.27	NE
	80+ yoa	91.37	NE
ZOE-50 HZ/su 1-dose^$^			
	50+ yoa	90.09	NE
ZOE-70+ HZ/su 1-dose^$^			
	70+ yoa	69.51	NE

yoa: years of age; HZ: herpes zoster; PHN: postherpetic neuralgia; HZ/su: herpes zoster subunit; NE: not estimated; ZVL: Zoster Vaccine Live; SPS: Shingles Prevention Study; ZEST: Zoster Efficacy and Safety Study

$ : Data on file

**Figure 6.**
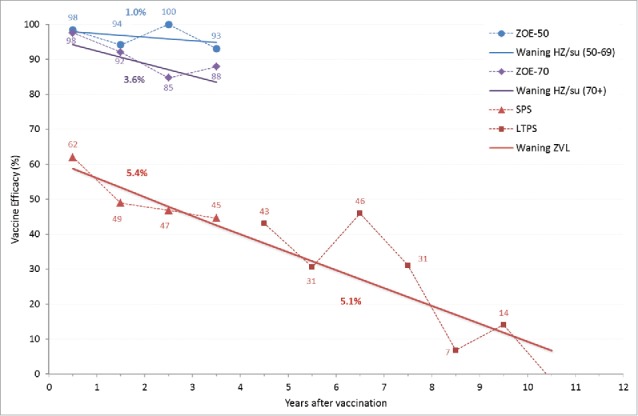
HZ efficacy waning over time. HZ: herpes zoster; SPS: Shingles Prevention Study; LTPS: Long Term Persistence Study.

The duration of protection for ZVL was modeled based on the data from the SPS and STPS/LTPS as presented by Morrison et al.^37^ and Oxman et al.^14^ (see Fig. 6 and supplemental text).^39^ The fitted line suggests a waning of approximately 5.4% per year during the first 4 y and 5.1% thereafter. In the ZONA model, the overall efficacy against PHN is assumed to wane at the same rate as efficacy against HZ which is consistent with the assumptions used in a previous German cost-effectiveness paper by Ultsch.^12^ Note in the ZONA model, both the HZ and PHN efficacy values used (i.e., age-specific efficacy at time 0 for the ZVL) are automatically adjusted to take into account the waning during the clinical trial follow-up period.

The efficacy of HZ/su, when administered as a 2-dose vaccine was evaluated in 2 phase III clinical trials in 16,161 individuals 50+ years of age (ZOE-50) and in 14,816 individuals 70+ years of age (ZOE-70), respectively,^17, 18^ that were conducted in parallel at the same study sites. Individuals 70+ years of age were randomly assigned to ZOE-50 or ZOE-70. This allowed pre-planned pooled analysis combining all individuals 70+ in both studies (referred to as ZOE-70+). The HZ efficacy in the ZOE-50 study is consistent across all age groups within the study (see Table 5). Similarly, the HZ efficacy for the age groups 70+ remains consistent across age groups in the ZOE-70+ pooled analysis.

To evaluate VE over time, a linear approximation was fitted to the yearly VE estimates derived from the ZOE-50 and ZOE-70+ analyses. Figure 6 presents the HZ VE waning over time for HZ/su. The VE estimate at time 0 (i.e., take) was 98.4% in ZOE-50 and 97.8% in ZOE-70+ analyses. The waning was approximately 1% in the ZOE-50 study and 3.6% in the ZOE-70+ analyses. Based on this data, it was assumed that for individuals aged 50–69 years, the HZ efficacy wanes at 1% annually during the first 4 y post-vaccination, at 2.3% during the subsequent years until the age of 69 and at 3.6% for all individuals aged 70+. Due to the very high efficacy observed against HZ in the ZOE-50 and ZOE-70 studies, the number of breakthrough cases was small, i.e., only 4 PHN cases (zero in the ZOE-50 and 4 in the ZOE-70 vaccination groups). The overall vaccine efficacies against HZ and PHN were similar within each study (see Table 5) and consequently no top-up VE against PHN was assumed for HZ/su.

As the HZ/su vaccine is developed as a 2-dose schedule, there was no pre-specified objective to assess 1-dose VE in the phase III studies. However for the public health impact analyses and future cost-effectiveness models, it is necessary to model 1-dose efficacy as well as a 1-dose waning scenario, as not every individual will receive 2 doses. In both the ZOE-50 and ZOE-70 studies there was a high compliance for the second dose, which limited the possibility to generate robust results for VE post dose 1. In addition, the mean follow-up period for individuals with one dose was 76 d for ZOE-50 and 85 d for ZOE-70+ analysis. The following 1-dose efficacy was observed: 90.1% (C.I.: 58.9%-98.8%) in ZOE-50 and 69.5% (C.I.: 24.9%-89.1%) in ZOE-70+. In the absence of long-term data on the waning of VE data for 1-dose scenario, it is assumed that the VE for 1-dose of HZ/su against both HZ and PHN wane at the same rate as that for the VE of ZVL (see Fig. 6).

It is assumed that the HZ/su vaccine doses are given 2 months apart. Coverage of the first dose was assumed to be 40% which is comparable to the influenza vaccine coverage in individuals aged 60+ in Germany.^21^ Compliance of the second dose of HZ/su is assumed to be 70% in the base case. Two scenario analyses were performed to explore the impact of using alternative compliance rates for the second dose of HZ/su, i.e., assuming 50% and 90%, respectively.

A DSA was conducted to assess the robustness of the results. The parameters modified in the DSA were: incidence rates, vaccine coverage, HZ/su second dose compliance, vaccine efficacy and waning of efficacy for both vaccines (the ranges are detailed in Table S1). Sensitivity analyses were performed deterministically, modifying the value of one base case parameter at a time and recording the corresponding number of HZ cases avoided. The results of the DSA are summarized in a tornado diagram.

A PSA was conducted to account for the full uncertainty in model inputs and to explore the impact on the outcomes (i.e HZ cases avoided). 5,000 Monte Carlo simulations were run, in which input values were simultaneously sampled from probability distributions. The ranges used for the PSA are consistent with the DSA analysis (detailed in the Table S1). All parameters were sampled across β distributions. Age specific incidence parameters which varied across age groups were assumed to be correlated using a correlation of 0.5. The results of the probabilistic sensitivity analyses are presented separately using a histogram displaying the HZ cases avoided with HZ/su compared with ZVL.

Scenario analyses were performed to explore the outcomes under specific scenarios. The input values varied in the scenarios considered are listed in Table S2. The variables included in the scenario analyses were: second-dose compliance for HZ/su, efficacy and waning of HZ/su for both 1 and 2-dose.

## Trademark section

Zostavax is a trademark from Merck Sharp & Dohme Corp.

## Supplementary Material

Supplemental_Material.zip

## Figures and Tables

**Figure 7.**
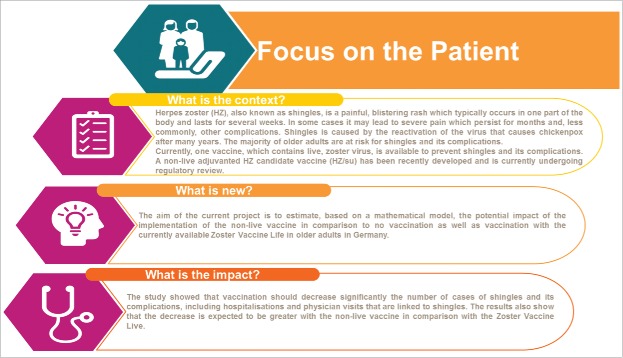
Focus on the Patient.
