# Supplementary material for: Assessment of the potential public health impact of Herpes Zoster vaccination in Germany
Source: Hum Vaccin Immunother. 2017 Jul 14;13(10):2213–21. doi: 10.1080/21645515.2017.1345399 (PMC5647993; doi:10.1080/21645515.2017.1345399)
Supplement: Supplemental_Material.zip [file khvi-13-10-1345399-s001.zip › Supplemental Material.docx]

Supplementary Material

Assessment of the potential public health impact of Herpes Zoster vaccination

in Germany

Desmond Curran, Desirée Van Oorschot, Lijoy Varghese,

Lidia Oostvogels, Tomas Mrkvan, Romulo Colindres,

Alfred von Krempelhuber & Anastassia Anastassopoulou

Human Vaccines & Immunotherapeutics

The persistence of protection following Zoster Vaccine Live (ZVL) was evaluated through longer-term follow-up of subjects in the Shingles Prevention Study (SPS) for 4 to 7 years post-vaccination in the Short-Term Persistence Study (STPS) and 7-11 years post-vaccination in the Long-Term Persistence Study ([LTPS](http://www.ncbi.nlm.nih.gov/pmc/articles/PMC4357816/)).^1, 2^ A concurrent placebo control was not available in the LTPS; data from prior placebo recipients were used to estimate vaccine efficacy (VE), as presented by Morrison *et al.*^2^ The fitted line suggests a waning of approximately 5.4% per year during the first four years. The waning from 5-11 years post-vaccination was estimated to be 5.1% (See Manuscript Figure 6).

As the follow-up in the Zoster Efficacy and Safety Study (ZEST) was approximately 1 year, the reported efficacy values in subjects aged 50-59 years represent the first-year efficacy.^3^ For the SPS study, the follow-up was approximately three years and as such, the reported efficacy is assumed to be the values at year 2 (i.e. at the midpoint of follow-up = 1.5 years).^1^ As such, the efficacy at year 1 is estimated as the efficacy at year 2 + the waning, e.g. for the cohort 60-64 the VE at year 1 (see manuscript Table 5) is 63.89 + 5.4 = 69.29.

In the absence of long-term data on the waning of VE data for 1-dose scenario, it is assumed that the VE for 1-dose of HZ/su against both HZ and PHN wane at the same rate as that for the VE of ZVL (See Manuscript Figure 6 and supplementary material Figure 1).

Based on the ZOE-50 and ZOE-70 ^4, 5^ clinical trial data, it was assumed that for subjects aged 50-70 years, the HZ efficacy for 2-doses of HZ/su wanes at 1% during the first four years post-vaccination, at 2.3% during the subsequent years until the age of 69 years and at 3.6% for all subjects aged 70+ years (See supplementary material Figure 2).

Supplementary material Figure 1: HZ/su 1-dose HZ vaccine efficacy waning over time for two different age groups

yoa: years of age; HZ: herpes zoster; HZ/su: herpes zoster subunit

Supplementary material Figure 2: HZ/su 2-dose HZ vaccine efficacy waning over time for three different age groups

yoa: years of age; HZ: herpes zoster; HZ/su: herpes zoster subunit

Supplementary table 1 presents the input parameters for the deterministic sensitivity analysis. A modified bootstrap sampling method was used to estimate the confidence intervals for some efficacy and waning parameters for both HZ/su and ZVL.^6^ The modified bootstrap sample uses the summary information reported in the clinical trial (e.g. treatment group, year, sample size, number of HZ cases, follow-up time) to generate samples representing the original study sample (size n). Sampling is carried out with replacement (size n). Thus some “subjects” in the original sample may be included several times, while others are excluded altogether. This creates a bootstrap dataset of the same size as the original study. A linear regression analysis is then fitted on this sample generating a point estimate (efficacy take) and slope (waning). This is done 1,000 times and all results are stored. One can then investigate the 95% confidence interval by sorting the observations and taking the 25^th^ and 975^th^ observations respectively.

Supplementary material Table 1: Input variables for the Deterministic Sensitivity Analysis

| **Parameters** | **Low Estimate** | **High Estimate** |
| --- | --- | --- |
|  | | |
| **Incidence (per 1,000)** | Schmidt-Ott et al. ^7^ | Hildebrand et al. ^8^  (upper 95% CI) |
| 50-59 yoa^$^ | 4.5 | 7.9 |
| 60-64 yoa | 6.3 | 10.3 |
| 65-69 yoa | 7.2 | 11.8 |
| 70-79 yoa | 8.5^$^ | 13.8 |
| 80+ yoa | 9.4 | 14.9^$^ |
|  | | |
| **Coverage** | Assumption | Assumption |
|  | 20% | 60% |
|  | | |
| **HZ/su second dose compliance** | Assumption | Assumption |
|  | 50% | 90% |
|  | | |
| **HZ/su 2-dose efficacy (Take)^*^** | Bootstrap Analysis | Bootstrap Analysis |
| 50-69 yoa | 95% | 100% |
| 70+ yoa | 94.1% | 100% |
|  | | |
| **HZ/su 1-dose efficacy (Take)^*^** | Data on file | Data on file |
| 50-69 yoa | 58.9% | 98.9% |
| 70+ yoa | 24.9% | 89.1% |
|  | | |
| **HZ/su 2-dose efficacy waning** | Bootstrap Analysis | Bootstrap Analysis |
| Years 1-4 | 2.6% | 0% |
| Years 5 until Age 69^#^ | 4.6% | 0.7% |
| Age 70+ | 6.6% | 1.4% |
|  | | |
| **HZ/su 1-dose efficacy waning** | Assumption | Assumption |
| Years 1-4 | 7.4% | 1% |
| After year 4 | 6.9% | 3.6% |
|  | | |
| **ZVL efficacy (PI)^9@^** | Prescription information | Prescription information |
| 50-59 yoa | 54.1% | 80.6% |
| 60-69 yoa | 56% | 71% |
| 70-79 yoa | 28% | 52% |
| 80+ yoa | 0% | 48% |
|  | | |
| **ZVL efficacy waning** | Bootstrap Analysis | Bootstrap Analysis |
| Years 1-4^#^ | 6.4% | 4.5% |
| After Year 4^#^ | 6.0% | 4.1% |

^$^ Data available for smaller age group but recalculated to fit age groups in the model

^*^ Take refers to efficacy at time 0

^@^ Data from the SPS study, reported in the Prescribing Information (PI). Note if the lower confidence interval was less than 0%, then 0% efficacy was assumed as the lower bound.

^#^ And assumptions

yoa: years of age; HZ/su: herpes zoster subunit; CI: confident intervals.

Supplementary material Table 2: Public Health impact of both HZ/su and ZVL under base case assumptions of 40 % coverage (HZ/su second dose compliance of 70%) over a lifetime horizon from the age of vaccination

|  | **Cases** | | | **Cases Avoided*** | | | | | |
| --- | --- | --- | --- | --- | --- | --- | --- | --- | --- |
|  | **No Vaccination** | | | **HZ/su** | | | **ZVL** | | |
|  | **50-59 yoa** | **60-69 yoa** | **70+ yoa** | **50-59 yoa** | **60-69 yoa** | **70+ yoa** | **50-59 yoa** | **60-69 yoa** | **70+ yoa** |
| **Subjects** | 12,993,405 | 9,533,940 | 12,968,294 | 5,197,362 | 3,813,576 | 5,187,318 | 5,197,362 | 3,813,576 | 5,187,318 |
| **HZ** | 4,478,667 | 2,528,890 | 2,208,714 | 725,223 | 533,162 | 486,794 | 198,477 | 196,000 | 104,640 |
| **PHN** | 787,035 | 484,125 | 446,385 | 113,218 | 98,456 | 98,121 | 26,313 | 36,053 | 55,462 |
| **Complications** | 548,637 | 309,789 | 270,567 | 88,840 | 65,312 | 59,632 | 24,313 | 24,010 | 12,818 |
| **Deaths** | 1,262 | 977 | 1,444 | 40 | 88 | 233 | 2 | 12 | 18 |
| **Hospitalisation** | 220,679 | 148,188 | 155,735 | 25,636 | 26,995 | 33,021 | 4,774 | 8,384 | 6,314 |
| **GP Visits** | 23,975,866 | 14,570,532 | 13,622,615 | 3,475,461 | 2,934,368 | 2,978,509 | 835,077 | 1,017,483 | 625,877 |

yoa: years of age; HZ: herpes zoster; PHN: postherpetic neuralgia; GP: General Practitioner; HZ/su: zoster subunit, ZVL: Zoster Vaccine Live.

*In vaccinated subjects compared to no vaccination over the life-time of the respective cohorts

Supplementary material Table 3: Public health impact scenario analysis assuming a coverage of 40% over a lifetime horizon from the age of vaccination (HZ/su second dose compliance of 50% and 90%)

|  | **Cases Avoided*** | | | | | |
| --- | --- | --- | --- | --- | --- | --- |
|  | **HZ/su : 50% second dose** | | | **HZ/su : 90% second dose** | | |
|  | **50-59 yoa** | **60-69 yoa** | **70+ yoa** | **50-59 yoa** | **60-69 yoa** | **70+ yoa** |
| **Subjects** | 5,197,362 | 3,813,576 | 5,187,318 | 5,197,362 | 3,813,576 | 5,187,318 |
| **HZ** | 607,754 | 468,921 | 421,903 | 842,693 | 597,403 | 551,684 |
| **PHN** | 93,054 | 86,021 | 85,007 | 133,383 | 110,891 | 111,236 |
| **Complications** | 74,450 | 57,443 | 51,683 | 103,230 | 73,182 | 67,581 |
| **Deaths** | 30 | 70 | 193 | 50 | 106 | 274 |
| **Hospitalisation** | 20,597 | 23,233 | 28,446 | 30,676 | 30,758 | 37,597 |
| **GP Visits** | 2,868,369 | 2,561,826 | 2,578,281 | 4,082,553 | 3,306,910 | 3,378,737 |

yoa: years of age; HZ: herpes zoster; PHN: postherpetic neuralgia; GP: General Practitioner; HZ/su: herpes zoster subunit

*In vaccinated subjects over the life-time of the respective cohorts

Supplementary material Table 4: Scenario Analysis Inputs and Results for HZ Cases Avoided comparing HZ/su and the ZVL

|  | **Second-dose compliance for HZ/su** | **Initial efficacy of HZ/su**  **(1-dose)** | **Initial efficacy of HZ/su**  **(2-dose)** | **Waning of HZ/su efficacy**  **(1-dose)** | **Waning of HZ/su efficacy**  **(2-dose)** | **HZ Cases Avoided** |
| --- | --- | --- | --- | --- | --- | --- |
| **Scenario 1** | ↑ | ↑ | ↑ | ↑ | ↑ | 2,522,181 |
| **Scenario 2** | -- | -- | ↑ | -- | ↑ | 1,967,254 |
| **Base Case** | -- | -- | -- | -- | -- | 1,246,062 |
| **Scenario 3** | -- | -- | ↓ | -- | ↓ | 629,732 |
| **Scenario 4** | ↓ | ↓ | ↓ | ↓ | ↓ | 220,854 |

↑=High Estimate, ↓=Low Estimate, -- Base-case value. Refer to Supplementary material Table 1 for high/low Estimates

HZ: herpes zoster; HZ/su: herpes zoster subunit; ZVL: Zoster Vaccine Live

**References:**

1. Oxman MN, Levin MJ, Johnson GR, Schmader KE, Straus SE, Gelb LD, Arbeit RD, Simberkoff MS, Gershon AA, Davis LE, et al. A vaccine to prevent herpes zoster and postherpetic neuralgia in older adults. N Engl J Med 2005; 352:2271-84.

2. Morrison VA, Johnson GR, Schmader KE, Levin MJ, Zhang JH, Looney DJ, Betts R, Gelb L, Guatelli JC, Harbecke R, et al. Long-term persistence of zoster vaccine efficacy. Clin Infect Dis 2015; 60:900-9.

3. Schmader KE, Levin MJ, Gnann JW, Jr., McNeil SA, Vesikari T, Betts RF, Keay S, Stek JE, Bundick ND, Su SC, et al. Efficacy, safety, and tolerability of herpes zoster vaccine in persons aged 50-59 years. Clin Infect Dis 2012; 54:922-8.

4. Lal H, Cunningham AL, Godeaux O, Chlibek R, Diez-Domingo J, Hwang SJ, Levin MJ, McElhaney JE, Poder A, Puig-Barbera J, et al. Efficacy of an adjuvanted herpes zoster subunit vaccine in older adults. N Engl J Med 2015; 372:2087-96.

5. Cunningham AL. The herpes zoster subunit vaccine. Expert Opin Biol Ther 2016; 16:265-71.

6. Hinkley DV. Bootstrap methods. J R Stat Soc Series B Stat Methodol 1988; 50:321-37.

7. Schmidt-Ott R, Schutter U, Simon J, Poulsen Nautrup B, von Krempelhuber A, Gopala K, Anastassopoulou A, Guignard A, Curran D, Matthews S, et al. Incidence and costs of herpes zoster and postherpetic neuralgia in German adults aged ≥50 years: a prospective study. J Infect submitted.

8. Hillebrand K, Bricout H, Schulze-Rath R, Schink T, Garbe E. Incidence of herpes zoster and its complications in Germany, 2005-2009. J Infect 2015; 70:178-86.

9. Prescription information Merck. Zostavax. Available at : http://www.merck.com/product/usa/pi_circulars/z/zostavax/zostavax_pi2.pdf [accessed 2017 September 25]
